# Supplementary material for: Temperature Incubation Influences Gonadal Gene Expression during Leopard Gecko Development
Source: Animals (Basel). 2022 Nov 17;12(22):3186. doi: 10.3390/ani12223186 (PMC9686823; doi:10.3390/ani12223186)
Supplement: Supplementary file 1 [file animals-12-03186-s001.zip › animals-1982853-supplementary.pdf]

## Supplementary Table S1: Nucleotide sequences identified by DDRT-PCR

| Clone name | Nucleotide sequence |
|---|---|
| **McAP2** | GCCCTTAAGCTTCGACTGTAGCTGCAACAGGAGCTAAGTAGCCATGGAGTGCCCAGCACCAGAGGCGT GTGCATGAGTGGAAGAAGAGGAACCAGCTGCGTACCACTGTTTCTGGTGGAGTCTTGTTATTGGGAAAT GATCGCACAGGCTGAGAGGTCACTAAGAGGCACCTGGAAGCTAGAAAAAGCACTAGGAAACTGCTAA GTATTATTTTAATGTGAGGACCAGCTGAAAGGGGCACTGGAGGCAGGGTTAGTCCCATTGCGGGAGCTC TCCTATACAAATGGAGAGGAAGGTTTGGGATGGCGGAGCCCTGTCATCTTGCCAGCACTCTGCCGTCTG TTCCCACCTGCCCACCTCCTGCATCTCTCTTCCCCCTACAAGCCTGTTTGGCTCTGCACATGAGCGTGAGC TGTAGCTTTGCAGTACTGGTTTGCTTGCTCCCTGCCTGCCCCCCTCCCCAGCCACATCCAAGTGCTGTGGG TAAGGCAGTATCTGTGGGGTAGGGACTGGCCTGTGTGTGTGTAGCTGTGGGTTCTTGAGATCTG |
| **Ma1AP5** | AAGCTTAGTAGGCAGTACTTGCTCACCCCTTTAAAGTTTCAGAAAAACAGTTCTCAAACGTGGCTGTTGT GTCATCTTTTTTTGGTACAGCCAAAGGAGAGTAGTGGAAATGAGTGTTTAGGGTGGGGGATCTTGCTTC AAGAGTAACTTGAGCTAAAGATTACCGCATGTTAGATAATGTAATGGGATGTAATTACACAAGGCAGAT TCAATATATGGTACTTAAGAGGGCTGAGCAGTGCAGTCGGTTGTTCCACTCAGTGCACTCCCACATCTTC AGCTGCTAAGAGCTGCTTGTACGCCTGTTGCAGTGTTTGTGTTTGTGTTCGAGTTGAATCATGTTTCCTGA AAGCACTGTCCTTTGGAAATCTGGATAAGTTACCTCCTGTAACAGGTGAATCAGGTTGTCAAGTACAACT TCGTACATCAATAAAGCAGAGCTCACTCCTAATAAAAAAAAGCTTAAGGGCAAATTCGTTTAAACCTGC AGGACTATTCCCTTTAGTGA GGGTTAATAATAATAACC |
| **Ma2AP5** | AAGCTTAGTAGGCCCCCTAGAATTAAGATTTTAGGATAAAAAGTGCACCATTTCTTTCTAAAACATCCA AGAAGATTTAAAACTACCTTTCCTCTGCTTCACACACTATTATTTTCCCTTCTAGAAAACTGATCTTTGTT ACTGTGTGAATGGAATCATCATTGCTCAGGAACACATTTAGTCTCTAGTTAGTGTTATTTGGGAAAACCA CAGGAGGCCAATCAGCGGGAAGAAGCATGGTATATACAAGCCAGTCTGTTGTAGCTGTAGACATGTTA ATTAATATATTTCTTCGACACCTAGGTTGATTGAAAAAGCAATTAAAGTGTAATTAAAATGTTTACTTCT GGTAGGAATGTCAACCAAGATGTGGAATATAGCAATTACCTATGACATTAGAGGAAGACGATGAGGTC TTTGAAGTGGTTCTGATCTCCCCTGTGAATGCTGTTCTTGGCACCAGGACAAAAGCTCAAGTGAAAATTT TGGACTCAAAAGGAGGTATTCTCTTTTGATCTTCATAATAAAATCTGGAGCCGTTTTGGCTGAGAGCTAC TTATTTTTCAGCACACAAATGTTGTTGCTGACACTTACCTTCAATCAAAATGCTTAATTTTATTTTAAAAA AAGAAAAAAATAGTCTTGACAGTTTCAG CCCTTTTCTCCTCTGGCATTCTGCATTCTTCTTCTGCTTAAGGAACC |
| **FaAP5** | AAGCTTTTTTTTTTATCCTTATTTACACTTCTAACTCCAGTACTAGCCTCAATACTCCCGCTAATAAAAA ATAAAACTTGAAAAAATAACACCTTAGCAATAAAACTAGCATTCTATATTAGTATTATACCAATATTAA TCTCTATTAATATAGGCTTACAATCAATAACAACAAATGCCATCTGATTTAATTCACATCTTAATATCAA AATTAGTTTACTATTTGACCAATACTCAATAATATTTCTACCAACAGCTCTATTTATCTCATGGGCTATCC TAGAGTTCACAAACTGATATATATCTGCAGATATTAACTTAGAACAATTTACTAAGTATTTAATAATTTTT TTACTCTCTATAATCACACTAACAACAGCAAACAGCTTACTACTACTATTCGTGGGGTGGGAAGGAGTA GGAATCATATCATTTCTACTAATCAGCTGATGATCCTCTCGTCTCAACGCAAACACAGCAGCCCTACAA GCAGTAATCTACAACCGCTTAGGAGACATTGGCCTATTTATAACTATAGCCTGATTTTCAATAAATCTTA ATACATTAGAAATACAACAAATATTCTCTTATAAAATAATCCCACTACTTCC |
| **McAP5** | AAGCTTAGTAGGCAGTTTAGCCGACCAGTGAGGCCTCCGCCTTTTTTCACCGTTAGAGTAGTGATTGTGG CGCCAACTTCTGAAGCAAGAAGAAAGCCTGGCGCGAGAAGGCATGCGAAGAGGCCTCGGCGAGGGGA AGAGACTTTGGGTGCAGGAAGGCAGCAAGTGAAGGAATGTGGGAGGCAGATCATTTCAATTGTTGTCA GGGAGAATGCCTGAATCAAAATTTCGGGAAGTCTTGCCTAAGCAAGGAAAGCTAGCAGTGGAAGATGT GACTACCATGGTGTTGTGCAAGCCGAAGTTGCTACCTTAAAATCAGTTACTTTAGAAAAGCTTGAGAAA ATGCAGCGTGAAGCACAGGAAACTATTAAACAGCAGGAACAGCTTGCTCAGCAGTCAGCACAATAATT CAGAAGCCATGTTTATCCACTATTTTATGTGCATAGATTTCTCCTGTTACTGTATTGGTGTAGATAGCAAA CAAATGTGGTATGAATAGTAAACTGAAAAGTGCTGTATTGTTAATCTTGGCTTATGTTCTTTATATGCAA AGACTTTAA AAATGATTTAATCCTGAAAAAAAAAAAGC |
| **Fg1AP6** | AAGCTTTTTTTTTTGTAAGGAATTCATAAATATTTATTATGTTTAACAGATGCAGCAACGATAATGATTT AAAAAAAAATGTAACAGCTGAAATGTTCTTCGCCTGGTCTCTAAACTGAAGACATAAATCCCTCTAGAA TCAGCATACCACCTTATTCTAATTTGAAGAGAGAGAGACAGAGACAGAGACAGAGAGATTGCAAAGTT ATGGGGAGGATGTATGTACGTATGTATTTACTAAAAATACAGAGGCCCACAAAATGATGAGAAATAAA CCCTGCTTGACATAAAAAGGAATCGAAAGAGCACCCTGGCGCATACACGTTGATTCCATTTCCTGACAG CATCAGAAATGCAAACGTCTGTACATCTCTCATCAGCAACAACCAGCTGCCACAACGGAGCTCCTATTC TCAACCCTTATACCGTCTCAGAATAAATTAGTTTGAAGATAGTTATGATGGTGCAAGCTTA |
| **FcAP7** | TAGCTTTTTTTTTTTCCATTACCAGTGGGTTTTGAAATTGCTTGCTCACAATGTCTTCTTTCATTTTTTTTGGT AGTAAACTGCACAATCATATCCCTAGGCAGATTTTTTTTGAGCTGCATATCTTGAATTAATTCTGTACGC CATGTCCACATATCTTGATCTCTTCCATGGTTTTATTCAAGAATTCTGCTAATATTTCCGATATCTGTTCTT GGGCTACCTTCCCCTCCTTCTCCGGAACGCCACGAAATCTATGTTGTCTCTCCATATATTTACATTCCATC ACCGCCACTCTTTCATTCAAGCCTGTGGTTTCTGCTTTAA ATGTAGTGGTTAGCTTCTCCGTCCTTTCTTCCACCTCGTTAAGCTTA |
